# Cancer Genetic Counselor Information Needs for Risk Communication: A Qualitative Evaluation of Interview Transcripts

**DOI:** 10.3390/jpm3030238

**Published:** 2013-09-03

**Authors:** Casey Lynnette Overby, Wendy K. Chung, George Hripcsak, Rita Kukafka

**Affiliations:** 1 Department of Biomedical Informatics, Columbia University, New York, NY 10032, USA; E-Mails: hripcsak@columbia.edu (G.H.); rik7001@dbmi.columbia.edu (R.K.); 2 Program for Personalized and Genomic Medicine and Center for Health-Related Informatics and Bioimaging, University of Maryland School of Medicine, Baltimore, MD 21201, USA; 3 Departments of Pediatrics and Medicine, Columbia University Medical Center, New York, NY 10032, USA; E-Mail: wkc15@columbia.edu

**Keywords:** cancer genetic counseling, information exchange, information needs, needs assessment

## Abstract

Personalized medicine is a model of healthcare that is predictive, personalized, preventive and participatory (“P4 Medicine”). Genetic counselors are an ideal group to study when designing tools to support cancer P4 Medicine activities more broadly. The goal for this work was to gain a better understanding of the information cancer genetic counselors seek from their patients to facilitate effective information exchange for discussing risk. This was an analysis of a qualitative data set from interviews of eight cancer genetic counselors, recruited from three institutions. Genetic counselors at each site were interviewed using a semi-structured, open-ended questionnaire. A selective coding approach was used to determine major themes associated with genetic counseling information needs for communicating risk. We generated a model for understanding categories of genetic counseling information needs to support risk communication activities. Common activities for risk communication included risk assessment and tailoring communication. Categories of information needs included: (a) clinical patient characteristics, (b) social and cognitive patient characteristics and (c) patient motivation and goals for the genetic counseling session. A logical next step is for this model to inform the design of software systems for pre-visit patient planning and delivering just-in-time educational information to facilitate cancer risk communication activities.

## 1. Introduction

Personalized medicine may be defined broadly as a model of healthcare that is predictive, personalized, preventive and participatory (“P4 Medicine”). [1] The use of genomics data for P4 Medicine is becoming more frequent, given the growing availability and decreasing costs of testing. Involving primary care physicians in delivering genetic services may be appropriate, given that the workforce of genetics professionals is limited in size. Primary care physicians generally have a favorable opinion of using cancer genetic data in their practice [2,3,4]; however, they do not feel adequately informed [5,6,7]. Effective genetic counseling makes complex genetic information understandable and personally relevant to the patients in terms of values, beliefs and lifestyle. Genetic counselors are among the few healthcare providers who are formally trained to deliver such complex genetic information and are, thus, an ideal group to study when designing tools to support counseling activities more broadly.

The body of literature investigating information exchange with patients from the perspective of genetic counselors is modest. Moreover, studies that do exist focus primarily on what and how information is conveyed to the patient and the influence on patient understanding [8,9]. This study, however, is concerned with genetic counselor information needs, so counselors can more effectively convey information to their patients. For example, previous work indicates that if a healthcare provider is able to address the patients’ main concerns when they give bad news about cancer, and the patient perceives information to be relevant to their situation, then consultation is more likely to be beneficial [10].

Information exchange is also described as the first two steps in shared decision making among patients and healthcare providers [11]; that is (1) both the patient and the provider are involved and (2) both parties share information. A 2012 review of key concepts relevant to shared decision-making demonstrates that much of this literature focuses on the direct needs of patients [12]. This further illustrates a need for more studies that investigate genetic counselor information needs. 

Such knowledge of information needs will be important for designing usable technologies. Needs assessments are commonly conducted to identify user needs that inform software system design and establish system components [13]. With the future goal of designing software systems to facilitate cancer P4 Medicine practices more broadly, we conducted a needs assessment study with experienced cancer genetic counselors. A subsequent study is in progress to understand how the information patients are prepared to give aligns with genetic counselor information needs.

## 2. Methods

### 2.1. Setting

This was a secondary data analysis of a previously collected qualitative data set from interviews of eight cancer genetic counselors recruited from Columbia University (New York, NY, USA), Huntsman Cancer Institute (Salt Lake City, UT, USA) and Fox Chase Cancer Center (Philadelphia, PA, USA). Informed consent was obtained for all participants, and each institution’s Institutional Review Board approved the study protocol. Data collection occurred over 3.5 months (late November through early March) in the year, 2010. The data were pooled and analyzed at Columbia University (protocol: IRB-AAAD6374).

### 2.2. The Interview

Cancer genetic counselors at each site were interviewed using a semi-structured, open-ended questionnaire. This interview included fifteen questions concerning communication processes (see Appendix 1). A team of a medical geneticist, a programmer and an informatics researcher developed questions. They were structured to gather data on the providers’ professional perception of risk communication, tailoring patient variables and patient communication procedures. The original goal was to use these data to identify common approaches to tailor risk communications and to determine differences in communication processes between genetic counselors and sites to inform the design of a tool for genetic counselors to support these processes. In this secondary analysis, our focus is narrowed to understanding the specific information cancer genetic counselors seek from their patients to support communication activities. Our ultimate goal is to inform the design of a tool that supports the exchange of information between patients and non-genetic counselor healthcare providers participating in cancer P4 Medicine activities. 

Each genetic counselor was interviewed once by telephone by one graduate-level research assistant. The duration of each interview was approximately 60 min. Audio recordings of each interview were assigned identification numbers and transcribed. We developed a codebook that initially included general themes extrapolated from the different categories of information elicited within the interview. Two graduate-level research assistants double-coded the transcripts. As new themes emerged, they were included in the codebook and assigned appropriately. Meetings were held bi-weekly to discuss discrepant coding among the research team. The NVivo 8 qualitative software program (QSR International; Doncaster, Australia) was used to maintain an electronic database of the consensus analysis of transcripts and information about each code. A final comparison of coding across all interviews yielded 67.75%–87.77% agreement.

A “selective coding” approach [14] was utilized to determine major themes associated with genetic counselor information needs for common activities (e.g., tailoring communication for the patient). The themes were confirmed for accuracy using member checking with one participant who was removed from the data set.

## 3. Results

### 3.1. Sample Characteristics

The demographics of the eight cancer genetic counselors interviewed are reported in Table 1. 

**Table 1:** Cancer genetic counselor demographics.

Characteristics	Genetic counselors (N = 8) N (%)
Gender	
Female	8 (100.0)
Male	0
Geographic location	
New York, NY	2 (25.0)
Salt Lake City, Utah	3 (37.5)
Philadelphia, PA	3 (37.5)
Education	
Master’s degree (M.S.), genetic counseling	8 (100.0)

### 3.2. Summary of Findings

**Figure 1:**
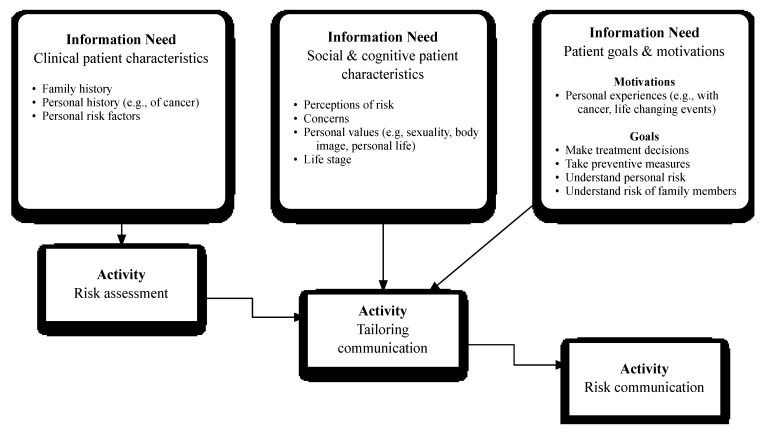
A conceptual model for genetic counselor information needs for risk communication.

Results indicated that participants engaged in genetic counseling exhibit three dimensions of information needs to support common activity categories. As shown in Figure 1, provider information needs include: (a) patient goals and motivation for the genetic counseling session, (b) social and cognitive patient characteristics and (c) clinical patient characteristics. 

The common activity categories were: (a) performing an assessment of risk, (b) tailoring communication for the patient and (c) risk communication with the patient. Member checking later revealed that these activities are related to one another. We therefore refined our model to reflect this relationship. Specifically, after performing an assessment of risk, tailoring occurs during risk communication with the patient. Thus, risk assessment and tailoring communication were considered common activities supporting risk communication. Common *risk assessment* activities discussed by the cancer genetic counselors in this study included assessing risk (e.g., risk for primary cancer, risk for recurrent cancer, risk of carrying a *BRCA1/BRCA2* mutation) and determining risk reduction with specific interventions (e.g., cancer surveillance, medication, surgery, radiation, lifestyle changes). *Tailoring communication* activities discussed by the genetic counselors included addressing patient concerns, assessing how the patient perceives risk, assessing how the patient understands numerical data, assessing anticipated reactions to possible options for action and communicating possible actions (e.g., cancer screening, cancer risk reduction, treatment, genetic testing). 

#### 3.2.1. Theme 1: Clinical Patient Characteristics

Cancer genetic counselors indicated that clinical patient characteristics (family history, personal history of cancer and personal risk factors) are important for risk assessment. Examples in which interviewees describe their process for collecting such data are as follows:

*“We send out what we call our health history questionnaire, and that’s many, many questions on your health and things that you’ve been exposed to. Now, it’s extremely comprehensive, because we’ve put together all of our risk assessment programs, so it has to do with tobacco exposure and alcohol exposure and a lot of different medication exposures, radiation exposures […]”*

*Subject 3*

*“On the medical history questionnaire, we ask about cancer diagnosis, as well as personal screening, when they started getting mammograms, if they’ve had breast MRIs in the past, how many breast biopsies they’ve had, enough information for us to do a Gail Model Risk if they haven’t had breast cancer themselves. …Then, with the family history questionnaire, if they return that ahead of time, our schedulers put that into Progeny, so we already have a full pedigree drawn out when we go into the session and we’re just kind of asking for updates or any changes if they learned more information about family members in the meantime.”*

*Subject 4*

These quotes also illustrate that relevant data may be collected prior to coming into the clinic. Risk assessment commonly involves use of risk assessment tools to assess risk for breast cancer (e.g., the Gail model [15]) or for carrying a genetic mutation (e.g., BRCAPro [16]). Several interviewees described this process:

*“So, we basically have most of the family history information when the patient comes in to see us; so based on that information, we can use various different models that we have to assess the likelihood that somebody may actually carry a mutation in either BRCA 1 or 2.”*

*Subject 7*

*“We will get family history information, and we can plug that into Progeny and BRCAPro. So, we can get those kinds of [risk assessment] numbers before somebody actually walks in the door.”*

*Subject 8*

After performing risk assessments, genetic counselors present risk information accounting for patient social and cognitive patient characteristics.

#### 3.2.2. Theme 2: Social and Cognitive Patient Characteristics

Understanding social and cognitive patient characteristics was a recurrent theme among cancer genetic counselors discussing risk communication activities. To better tailor communication, cancer genetic counselors commonly ask questions to elicit from their patients personal values, such as issues of sexuality (e.g., how do they feel about their breasts in terms of their femininity and sexuality?) and where they are in their life stage (e.g., do they want to have children in the next five years?). These questions help providers to assess patient priorities and concerns, which can influence how they tailor a counseling session to an individual. One study participant, for example, described two patients she counseled a week earlier who were both newly diagnosed with breast cancer. One patient was advised to have a mastectomy and decided to have genetic testing first to refine her risk for a new breast cancer diagnosis, in the hopes that the risk was low to justify not having a mastectomy. The other patient was advised to have a lumpectomy and radiation and decided to have a bilateral mastectomy to ensure that she would never have another breast cancer diagnosis.

*“[…] for one, it was her body image, and to the other one, it was her fear of getting a new breast cancer. So, even though numerically the risk of getting a new breast cancer for both was probably 25%, the way they put that together was totally different.”*

*Subject 3*

For a patient who already believes she is at high risk, it may be important to tailor communications to emphasize what is currently known about their risk and what a genetic test result can and cannot add to refining their risk. An interviewee describes how they tailor communication of treatment options given a patient has a highly penetrant mutation that increases their risk for breast cancer:

*“I try and get a sense from them if they’ve thought ahead of time, you know, if they did come back positive, what management would they be comfortable with. Would they go more towards screening or would they go more towards surgery. And for all the patients, regardless of what their response is, I do present surgery as an option, but I definitely emphasize in all cases that it’s an option and that it’s not a strict recommendation to get it. If they are very much saying they wouldn’t consider surgery, I wouldn’t go into as many details about different surgical options or different cosmetic outcomes or whatnot.”*

*Subject 4*

Cancer genetic counselors also want to understand the patients’ perceptions of risk and how the patient understands numerical data in order to tailor risk discussions. In response to the question: “What factors do you think may lead to misunderstanding about risk and how do you address these factors?” one interviewee describes this process as follows:

*“I think it’s important to figure out what the patient initially perceives their risk to be. Whether they indicate that numerically or, you know, with words like high or low - so, you know where they’re coming from [and] so that you can present your risk information in the appropriate context. I think that’s figuring out how they’re perceiving things. If they’re overestimating, underestimating, maybe estimating correctly, but not necessarily assigning the right numerical value to, it helps you know what you need to present to them to hopefully get them to a point of more accurate understanding.”*

*Subject 6*

In addition to assessing how the patient understands risk, cancer genetic counselors also want to know how the patient understands numerical data. Different ways to describe risk (e.g., percentage risk *vs*. category of risk) may be interpreted by patients in different ways. In response to the question: “How do you verbally tailor the information you give the patient? Do you phrase or state risk in a specific way?” one interviewee says:

*“[If] I’m getting the sense that they’re not as savvy [with numbers] as some of the patients, I really try to make it less number-based and just try to help them understand the overall concept that, you know, something increases* versus *decreases risks. Then, I have some patients that are just really savvy and have really great questions, and then, I’ll get into much more detail with them. So, I’ll kind of let the patient give me the cues to help me tailor.”*

*Subject 5*

It is common for genetic counselors to deal with variation in how patients perceive risk by describing information in several different ways. For example, one interviewee said:

*“I think people need to hear the information in several different ways, so increased risks, you know, decreased risks, no risk or population risk of breast cancer, that can mean very different things to people.”*

*Subject 2*

If a genetic counselor is able to clearly characterize how the patient understands risk and numerical data, however, it may better facilitate tailoring risk discussions. For example, one interviewee describes how she communicated risk using descriptive terms, rather than absolute numerical risk:

*“I had a patient last week that said ‘I don’t think numbers mean anything to me. Numbers can lie, they don’t mean anything.’ So, for that person, I really tried to tailor that session to not be just about let’s look at this bar graph and let’s talk about risk, but let’s talk about what you think […], in genetic counseling we’re told to avoid being subjective, like [using] subjective terms, like low, medium and high. But, for somebody who can’t really grasp or doesn’t like numbers, you have to really categorize these risks for them.”*

*Subject 9*

After presenting risk information to the patient, genetic counselors tailor options for testing and intervention. Social and cognitive patient characteristics may be important to consider when tailoring these options. One interviewee, for example, describes their encounter with one patient for whom body image was an important factor when considering risk reduction interventions:

*“[…] I had a patient tell me that she’s had cancer and there’s no way that she’s ever going to be cut, period, and she told me how she was in labor for three days, and they wanted to do a C-section and there was no way she was going to let them give her any kind of scar on her body. So, from there, I learned how important her body image was to her. [After hearing that] I’m really not going to spend that much time talking about risk reducing surgeries, such as prophylactic mastectomy. […] We could focus on something else.”*

*Subject 9*

Although social and cognitive patient characteristics are important for tailoring options for testing and intervention, these decisions are made primarily based on patient goals and motivations.

#### 3.2.3. Theme 3: Patient Goals and Motivations for the Genetic Counseling Session

Cancer genetic counselors indicated that it was common for them to start counseling sessions with questions, like “What brought you here today?” “What are you hoping to learn?” “What do you want to get out of this session?” and “What are your experiences with cancer?” to elicit patient motivations and goals for genetic counseling sessions. Understanding patient motivations and goals was a recurrent theme in our participants’ responses to questions about risk communication activities. 

Eliciting patient motivations and goals is important for cancer genetic counselors to tailor options. Concerns may be in regard to risk of primary cancer, risk of recurrence or risk of second primary (e.g., ovarian cancer after breast cancer). Other concerns may be about children or other family members’ risk of getting cancer. The following quote illustrates the variability in the motivations and goals of patients that should be considered in risk communications:

*“[…] my patients come in for several different reasons. I’ll see patients who are newly diagnosed with cancer, and they’re trying to make treatment decisions, so they want to know how likely it is that their cancer is hereditary and how aggressive they should be in their treatment decisions. That’s one avenue of patients. I have another avenue of patients who have never been diagnosed with cancer, but have a strong family history of cancer, and they want to find out how likely it is that the cancers in their family are hereditary cancers. And, what they can do, what steps they can take to protect themselves against getting these cancers. And then, I have other people who come in because they’ve been diagnosed with cancer in the past and are now ready to deal with whether or not the cancer is hereditary. And maybe, they want to provide helpful information for other family members or children, siblings.”*

*Subject 3*

Tailoring the discussion to the risk of concern for the specific patient at that specific time is particularly important for determining appropriate treatment and prevention options for various scenarios. It was common, for example, for these interviewees to describe how patients’ personal experiences with breast cancer can lead to misunderstanding about risk. One interviewee describes how personal experiences with breast cancer influences perception of risk:

*“I don’t think when people are thinking about their risk, they’re thinking about them in numbers the way we are, so as geneticists, we’re thinking about your risk as 15 percent* versus *10 percent, but for the average woman, they might just be feeling like, ‘Yeah, my mom and sister got breast cancer; it’s probably going to happen to me too.’”*

*Subject 6*

Further, many of our study participants suggested that understanding strategies for risk reduction is a common goal that is important for tailoring communication. Treatment decisions (mastectomy *versus* lumpectomy), for example, are often the immediate focus of discussion for patients with newly diagnosed breast cancer.

It also appears to be important to understand patients’ personal experiences that influence their perceptions of risk. The following quote illustrates patient concerns about environmental effects, which should be addressed during discussions about risk.

*“[…] if they’ve had a lot of neighbors that grew up getting cancer, they could be really worried about the environmental effects. And, some people do live in areas where there are certain higher incidences of cancer, but oftentimes, they can consider any cancer to be greatly inflated with that. Where, at least with some of the studies, more cancers can be prone to [environment] than others. And, some of the cancer in the family may not be related to the environmental effects as much or just be very difficult to prove. So, [someone] who may be a good genetic testing candidate may still be focused on the environmental effects.”*

*Subject 4*

## 4. Discussion

This study is a qualitative investigation of cancer genetic counselor information needs. Genetic counselors complete common information gathering activities, including: (a) clinical patient characteristics, (b) social and cognitive patient characteristics and (c) patient motivation and goals for the genetic counseling session. Based upon these data, they assess risk and tailor communication, which are important for discussing risk. We confirm risk communication activities and information needs identified in other studies. The main contribution of this study, however, is a conceptual model for how information is gathered from patients to support risk communication. This model can inform the design of technologies for pre-visit patient planning and delivering just-in-time educational information to support cancer risk communication activities.

Risk communication activities are among commonly investigated genetic counseling processes [17]. In support of our findings, previous work indicates that counselees’ motivations for genetic counseling are often a topic of discussion [18]. Patients often have goals prior to a session [19] that could be incorporated into a consultation planning tool to help patients prepare for their counseling session and to help ensure that the session is tailored and most useful for the patient. A previous study suggests that insight into patient pre-visit needs may help counselors to better address patients’ concerns [20]. Following that study, however, investigators found that communications between counselors and counselees does not appear to reflect counselees’ pre-visit needs [18]. That finding further motivates exploring the development of tailoring technologies to facilitate the discussion of patient pre-visit needs, so that healthcare providers can respond more appropriately to patient-specific issues when counseling patients. 

Our conceptual model may inform the design of tailoring technologies delivered prior to a genetic counseling session. There are already examples in which use of consultation planning tools facilitate more client-specific counseling sessions [21] and improve patient satisfaction with counseling sessions [22,23]. These studies suggest that pre-visit patient planning can satisfy patient information needs and improve genetic counselor-patient communication. Our study provides evidence that genetic counselor information needs may also be satisfied prior to the counseling session. For example, we illustrated that clinical characteristics, such as family history and the patient’s medical history, can be gathered and risk calculated prior to the counseling session. 

Tailoring technologies may also be appropriate for delivering just-in-time educational information during “teachable moments” occurring within counseling sessions. Genetic counselors from this study, for example, indicate dealing with variation in how patients perceive risk by describing information in several different ways. Tailoring technologies may facilitate presenting numerical risk data in ways that account for such variation. Such technologies would be particularly useful for healthcare professionals who are not very familiar with genetics and are not formally trained to tailor risk discussions, but who are involved in the delivery of genetic services.

### Limitations and Implications for Future Research

To inform the design of a system to support cancer risk communication activities, we were able to gather rich descriptive data associated with genetic counseling processes through interviewing. Our findings, however, may be limited by recall bias. To ensure that eight counselors were sufficient to reach saturation of our model, we completed additional steps to confirm themes for accuracy using member checking, and we discuss our findings in relation to observational studies that investigate genetic counselor communication processes. Like most qualitative studies, the generalizability of the findings is limited to the group studied. Even so, our goal was to understand the experiences of this particular group of participants so we can design software systems to support cancer P4 Medicine practices more broadly.

The limitations of this study can serve to inform future studies. For example, the risk communication activities we identified are specific to cancer genetic counseling primarily testing for BRCA1/2 mutations known to increase risk for breast and ovarian cancer. The information needs we identified, however, may generalize to the perspectives of others who perform patient risk assessment activities for other diseases (e.g., cardiac or neurologic diseases) and based upon results from different tests (e.g., panel gene testing). Future studies should include multiple clinical specialties and tests in an effort to better understand the information needs that could be specific to disease areas and the ways in which test results are reported.

This work also highlights a need to confirm under which contexts genetic counselor information needs occur. For example, while we found that cancer genetic counselors want to understand patients’ perceptions of risk in their discussions, we were unable to delineate at what point this information is most important. Previous work suggests that risk perception does not appear to be a major subject in initial visits, but deferred until risk can be better stratified based upon the results of genetic testing [18]. In addition, of all of the studies identified in a 2008 review article, all but one assessed the initial consultation of clients [17]. This further indicates a need for more research on the exchange of information over time.

## 5. Conclusions

From this study, we have defined a conceptual model illustrating the dimensions of genetic counselor information needs and collecting specific types of information from patients during risk discussions. Types of information include (a) clinical patient characteristics, (b) social and cognitive patient characteristics and (c) patient motivation and goals for the genetic counseling session. Our model can inform the design of technologies for pre-visit patient planning and delivering just-in-time educational information to support cancer risk communication activities.

## Appendix

**Appendix 1:** Interview questions proposed to cancer genetic counselors.

Do you consider a discussion about risk important when you counsel your patients? When you discuss patient’s risk, is it risk for gene mutation or risk for breast cancer? Are you comfortable in discussing risk? Probe answer. What factors do you think may lead to misunderstanding about risk and how do you address these factors? What are you most interested in learning about your patient so you can then do a better job tailoring your counseling session to their specific needs and characteristics? How do you assess patient’s characteristics in order to tailor the counseling session? Is there a pre counseling assessment (questionnaire)? Can you customize the questions that are on that assessment? How do you verbally tailor the information you give the patient? Do you phrase or state risk in a specific way? Would a computer-based educational resource be useful in communicating with a patient before, during or after the counseling session? Probe: for example, before to do the assessment of psychosocial, medical and clinical patient characteristics. What types of information, other than risk, would benefit a patient, or a counselor in communicating with a patient, in making a decision on genetic testing? Do you provide a patient with information prior to the patient entering the clinic? What type of information and in what format?What type of literature do you give the patient when she/he enters the clinic? Do you send any information to the patient after the counseling session (example: a summary letter)? When does a patient decide to go ahead with the genetic test? Do the patients need to make a decision before or after they leave the clinic? What is the amount of time scheduled for a counseling session with a patient? What is the amount of time given for patient questions/follow-up discussion? Can a patient bring one (or more) companion(s) to the counseling session? Does the presence of other people affect the manner in which a counselor counsels a patient?
